# Socio-demographic profiles and obstetrics outcomes of pregnant women with epilepsy in a vulnerability State, Brazil

**DOI:** 10.1371/journal.pone.0271328

**Published:** 2022-07-20

**Authors:** Magnúcia de Lima Leite, Tatiana Natasha Toporcov, Janise Dal Pai, José Claudio da Silva

**Affiliations:** 1 Universidade Estadual de Ciências da Saúde Alagoas (UNCISAL), Maceió, AL, Brazil; 2 Universidade de São Paulo (USP), São Paulo, SP, Brazil; 3 Universidade Pontifícia Católica do Rio Grande do Sul (PUCRS), Rio Grande do Sul, RS, Brazil; 4 Faculdade de Medicina do Centro Universitário (CESMAC), Maceió, AL, Brazil; Columbia University Medical Center, UNITED STATES

## Abstract

**Introduction:**

The socio-demographic profile of pregnant women in low- and middle- income countries is characterized by low educational attainment and unemployment, leading to social and economic morbidity. characterized by limited opportunities for education, employment, and marriage, which are strongly related to the stigmatization of the disease. The study of the socio- profile and obstetric outcomes of pregnant women with epilepsy in Alagoas, Brazil, may help understand this scenario and facilitate the development of public policy strategies to reduce local morbidity.

**Objectives:**

We aimed to describe the sociodemographic profile of pregnant women with epilepsy and obstetric outcomes in Alagoas, Brazil.

**Methods:**

This cohort study was based on medical records of pregnant women with epilepsy in Brazilian high-risk maternity hospitals from 2008 to 2020. The following data were collected: age, race, education, marital status, occupation, number of pregnancies, delivery, and abortion. The inclusion criteria were pregnant women with and without epilepsy (control group) aged < 40 years.

**Results:**

The prevalence of PWWE was 0.49% (n = 224/44,917). Cesarean delivery was more frequent in PWWE than in pregnant women without epilepsy (adjusted odds ratio [OR] = 22.0; 95% confidence interval [CI] = 14.35–33.73; *p*<0,01). Abortion was associated with PWWE (OR adjusted = 1.72; 95% CI = 1.13–2.61; *p* = 0.01). Pregnant women in the countryside were more likely to develop epilepsy than those born in the capital (OR = 1.55; 95% CI = 1.12–2.14; *p* <0.01).

**Conclusion:**

The PWWE socio-demographic profile of the Alagoas had a predominance of brown- colored skin, single status, homemakers, and illiteracy with a high proportion residing in the interior of the state. The obstetrics data show a higher incidence of cesarean deliveries and miscarriages.

## Introduction

Epilepsy is a disorder characterized by the permanent predisposition of the brain to the generation and propagation of epileptic seizures (International League Against Epilepsy) [1], which affects 65 million people worldwide [2]. The incidence and prevalence of epilepsy are higher in low- and middle-income countries. Its lifetime prevalence for developed countries is 5.8 per 1,000, while in rural areas of developing countries it is 15.4/1,000 [3]. The incidence of epilepsy is 45/100,000/year in high income countries, compared to 81.7/100,000/year in low- and middle-income countries [4]. The differences in prevalence and incidence among these countries are explained in part by risk factors such as head trauma, central nervous system infections, and perinatal injuries, which are more common in poor regions, particularly in rural areas [5]. The global mortality rate among people with epilepsy (PWE) in high-income countries is two to five times higher than in the general population, whereas in low-income countries, it may be up to 37 times higher, especially among young people aged 10 to 29 years [6].

Epilepsy is the second most common neurological disorder during pregnancy, only behind migraine [7]. European studies indicate that 0.4% to 0.8% of pregnant women have epilepsy [7, 8] and that 95% of them undergo treatment with antiepileptic drugs (AEDs) [7]. Approximately a quarter of women with epilepsy (WWE) are in childbearing age [9] and may present a risk between 1 and 1.5 times higher when compared to women from the general population [7]. However, for maternal mortality, these rates may be 10 times higher among pregnant women with epilepsy (PWWE) [10]. Preeclampsia, premature birth, fetal growth retardation, and fetal and maternal mortality are among the most common complications. However, studies show that these complications are not frequent and do not support the contraindication of pregnancy, as more than 90% of WWE have normal pregnancy [7] thanks to adequate monitoring, guidance, and optimization of AED treatment [9–11]. In contrast with Europe, in Brazil, 40.5% of hospitalizations of PWWE seem to be related to their epileptic condition, while 16.9% correspond to the hospitalization of non-pregnant women and men with epilepsy [12]. In this regard, the literature points out that efforts should be focused on optimizing the pre-pregnancy health conditions of WWE [9–11], suggesting that in this period, they should be guided toward the optimization of seizure control. Studies shows that the frequency of seizures does not seem to change in most pregnant women, while in others it may increase between 15%–37% [13]. Furthermore, one study showed that pregnant women without episodes of seizures in the 9 months preceding conception have a probability of between 84% and 92% of remaining seizure-free during pregnancy [14]; in line with this; another study, performed in 1,297 PWWE, observed that the occurrence of epileptic seizures in the month prior to conception was the main predictor of relapse during the gestational period [15]. Thus, considering that most PWWEs require long-term treatment for seizure control, drug screening and adverse effects are essential to define an effective regimen with less teratogenic effects [9].

Stigmatization caused by PWE is one of the main factors affecting the quality of life of patients and their families, particularly in low- and middle-income countries [16].

In the past, epilepsy was attributed to supernatural causes and was the reason why many patients were banned from society. However, in the 1990s, denial or annulment of marriage due to epilepsy was a common practice, and people would consider that PWE should not be employed [17, 18]. PWE deal with the stress of living with a chronic unpredictable disease and, among other difficulties, suffer from discrimination, misunderstanding, and social stigma [2–19]. Research shows that their socio-demographic profile is characterized by limited opportunities of education, employment, marital prospects, and decreased self-esteem, which in turn may affect their social lives [20, 21]. In Brazil, studies show that the consequences of stigmatization among PWE are still present [12]. Although only a few studies have targeted the Brazilian population with epilepsy, a study from Santa Catarina state outlined the epidemiological profile of this sector; the authors observed that PWE were characterized by high levels of illiteracy (30.2%, n = 13) and had a stable union or were married (44.2%, n = 19) [22].

One systematic review and meta-analysis of observational studies included 38 studies with 2,837,325 pregnancies to assess the risks of maternal and fetal complications (excluding malformations and cognitive-behavioral deficits) regarding obstetric outcomes in PWWE versus pregnant women with no epilepsy (PWNE). This review indicated an increased risk of miscarriage, antepartum hemorrhage, postpartum hemorrhage, hypertensive disorders, induction of labor, cesarean section, and preterm delivery before 37 weeks, including spontaneous abortion [23]. The incidence of cesarean section has increased worldwide. A diagnosis of epilepsy and exposure to AEDs was significantly associated with an increased risk of induction of labor and cesarean section [24]. The cause of cesarean sections in WWE is multifactorial. Despite representing a noteworthy disease, epilepsy is not an indication for a cesarean section. Cesarean delivery is indicated in epilepsy patients with frequent seizures, those who have seizures during labor, those who cannot cooperate during delivery, and those whose seizures are provoked by physical activity [25–27]. Per the Brazilian culture concerning their preference for cesarean section delivery, social, institutional, financial, and obstetric practice factors influence their choice [28]. A retrospective study in 20% of community hospitals in the United States and a recent study in China observed that PWWE were more likely than PWNE to have a cesarean delivery at 40.5% vs. 33.1%; 85.6% vs. 50.3%, respectively [10–25].

There is limited information on the PWWE population in Alagoas, Brazil. Knowledge and understanding of the social context and obstetric outcomes would help promote public policies to improve their quality of life and raise the awareness of the general population about this chronic disease.

This study aimed to determine the prevalence, sociodemographic profile, and obstetrics outcomes in PWWE who received care at high-risk pregnancy reference centers in Alagoas. We hypothesize that the prevalence of PWWE is high in this state and that the sociodemographic profile is characterized by conditions of social vulnerability and adverse obstetric outcomes, as such conditions are common in northeast Brazil. Furthermore, this study aims to propose better public policies and highlight the need for health professionals to adopt strategies to prevent this outcomes.

## Methods

The present study was approved by the ethics committee of the University of São Paulo, São Paulo, Brazil (Number 4,604,088) and in the co-participating institutions, University of Health Sciences and Federal University of Alagoas (UFAL) (Numbers 4,491,415 and 4,422,629, respectively). This was a retrospective cohort study, with collected data from the physical and electronic medical records of PWWE and PWNE who received care from 2008 to 2020. The following data were collected: age; the official categories according the brazilian Census for race: white, black, yellow, brown and indigenous; Education: illiteracy (neither read, nor write), fundamental complete or incomplete, high school complete or incomplete, graduation complete or incomplete; Marital status: single, married, stable union, divorced or widow; Occupation: student, housewife and others employment; number of pregnancies; delivery and spontaneous abortion.

The participants were exempt from signing the consent form, and their anonymity was guaranteed. Our sample consisted of PWWE and pregnant women with no epilepsy (PWNE) younger than 40 years. Data collection was performed at the Prenatal and Prepartum Clinics of the University Hospital Professor Alberto Antunes—HUPAA- UFAL and Maternity School Santa Mônica, in Maceió, capital of Alagoas; Espaço Nascer Prenatal Clinic, in Arapiraca; and Dr. Clodolfo Rodrigues de Melo Regional Hospital, Santana do Ipanema, located in the countryside of Alagoas, Brazil. All these institutions are considered references in the care of PWWE in the Alagoas state. Both groups of pregnant women were recruited from the same high-risk referral centers. The total number of pregnant women in each center was recorded for prevalence calculation. For the confirmation of the diagnosis of epilepsy, the International Classification of Disease (ICD-10) codes G40.0–G40.8 were used, and our control healthy pregnant women were selected from the medical records. For descriptive analyses, categorical variables are presented as frequencies, and continuous variables as means and standard deviations. Chi-square tests and multivariate logistic regressions were performed to verify the association of the diagnosis of epilepsy with variables related to pregnancy and sociodemographic profiles. The crude and age adjusted ORs and their respective 95% confidence intervals (CIs) are presented. To verify the differences between groups for continuous variables, a t-test for independent samples was performed. A value of α equal to 5% was adopted for all analyses, which were conducted using the statistical software R v 3.6.1 (R Foundation for Statistical Computing, Vienna, Austria).

## Results

The PWWE comprised 0.49% (n = 224/44917) of high-risk pregnancy referral centers in the Alagoas state. The total amount of our sample was 716 pregnant women, with 31.2% for PWWE (n = 224) and 69.1% for PWNE (n = 492), aged 24.94 ± 6.25 and 23.98 ± 6.89 years, respectively (p = 0,07) (Table 1). Most pregnant women born in the countryside were more likely to have epilepsy than pregnant women who were born in the capital Maceió (adjusted OR = 1.57; 95% CI = 1.14–2.18; *p* <0.01) (Table 2). The majority of pregnant women with and without epilepsy were brown (n = 195/220, 88.6%; and n = 425 / 484, 87.8%, respectively), followed by white PWWE (n = 15/195, 6.8%) and PWNE (n = 47 / 484, 9.7% *p* = 0.17) (Table 1).

**Table 1:** Comparison of average socio-demographic and obstetric outcome variables between pregnant women with and without epilepsy.

Variables	Pregnant with epilepsy	Pregnant without epilepsy	*p*-value
	(n = 224)	(n = 492)	
**Age**			0.07
Mean and standard Deviation	24,94 (± 6.25)	23.98 (± 6.89)	
**Total**	220 (100%)	491(100%)	
**Race/color**			0.17
White (n = 62)	15 (6.8%)	47 (9.7%)	
Black (n = 22)	10 (4.6%)	12 (2.5%)	
Brown (n = 620)	195 (88.6%)	425 (87.8%)	
Total	220 (100%)	484 (100%)	
**Previous births**			0.58
Primipara (n = 292)	87 (39.7%)	205 (41.9%)	
Multiparous (n = 416)	132 (60.3%)	284 (58.1%)	
Total	219 (100%)	489 (100%)	

**Table 2:** Analysis of crude and age-adjusted odds ratio of sociodemographic variables of pregnant women with epilepsy.

Variable		OR crude		OR adjusted by age		
	OR*	95% CI	*p*-value	OR adjusted	95% CI	*p*-value
**ORIGIN**						
Maceió	Reference	-	-	Reference	-	-
Countryside	1.27	0.82; 1.99	0.27	1.57	1.14; 2.18	<0.01
**Race/color**						
White	Reference	-	-	Reference	-	-
Black	3.89	0.93; 16.13	0.06	2.49	0.90; 6.95	0.08
Brown	1.01	0.45; 2.21	0.98	1.36	0.74; 2.51	0.31
**Education**						
Illiteracy	0.81	0.30; 2.14	0.66	2.56	1.19; 5.51	0.01
Fundamental complete or incomplete	Reference	-	-	Reference	-	-
High school complete or incomplete	1.07	0.66; 1.73	0.79	1.17	0.83; 1.66	0.36
Graduation complete or incomplete	1.38	0.33; 5.84	0.65	3.77	1,20; 11.84	0.02

However, no association was found between PWWE and brown ethnicity (adjusted OR = 1.36; 95%CI = 0.74–2.51; *p* = 0.31) (Table 2).

**Fig 1:**
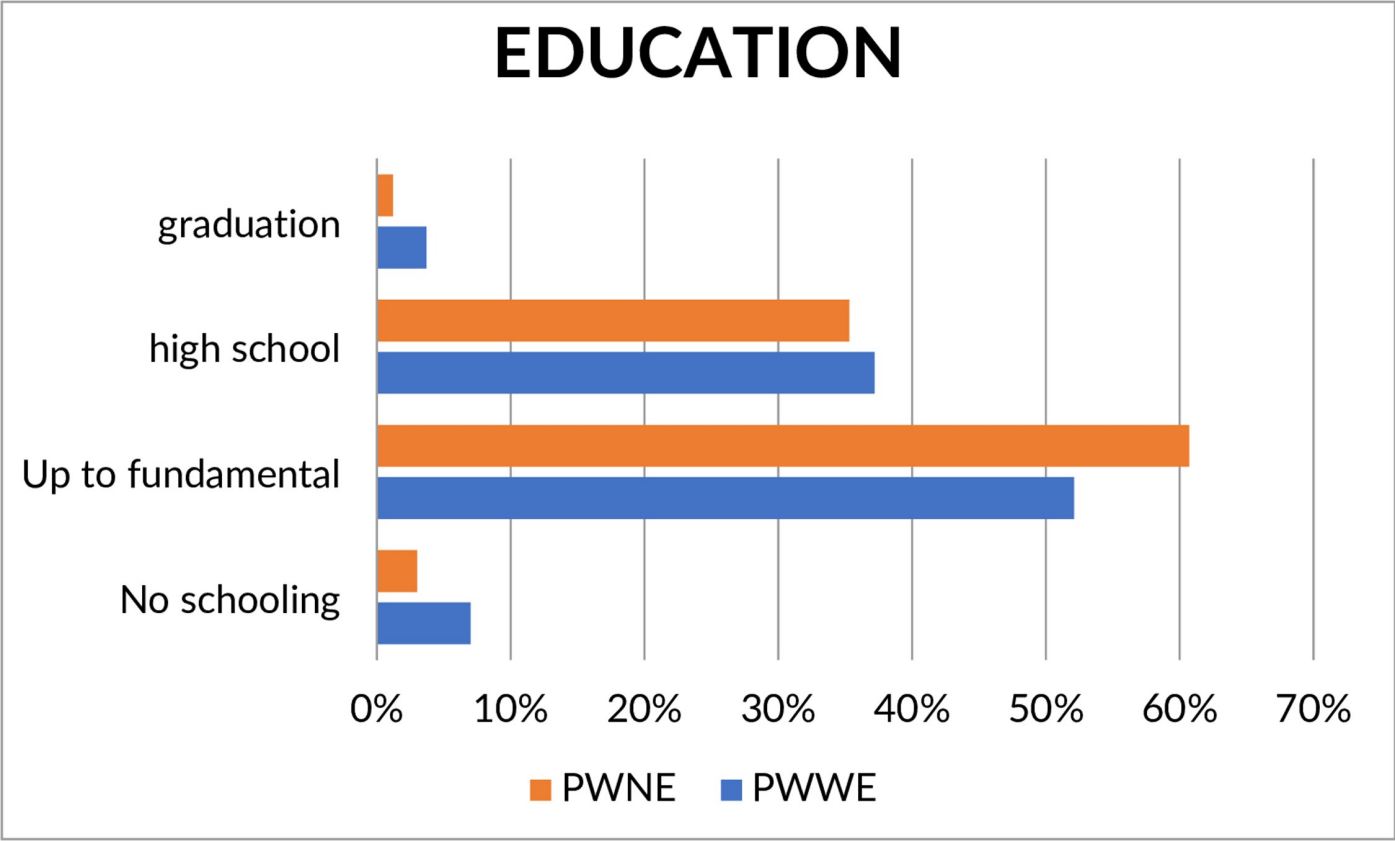
Comparison of average in education variables between pregnant women with and without epilepsy. The figure shows the proportion of pregnant women without epilepsy with complete or incomplete primary education and that of pregnant women with epilepsy that were illiterate, had complete or incomplete high school level, and complete or incomplete graduation level.

The results revealed higher levels of illiteracy in PWWE (n = 7/215, 15%) than PWNE (n = 14/468, 3%), while incomplete or complete elementary school was observed in 52.1% (n = 112/215) and 60.7% (n = 284/468) of PWWE and PWNE, respectively. Similarly, incomplete or complete high school was achieved by 37.2% (n = 80/215) of PWWE and 35.3% (165/468) of PWNE, and higher education was achieved by 3.7% (n = 3/215) of PWWE and 5.1% (284/468) of PWNE (Fig 1 and S1 Table). However, an association of illiteracy and PWWE was observed only after adjustment for age (adjusted OR = 2.56, 95% CI 1.19–5.51, *p* = 0.01) (Table 2).

**Fig 2:**
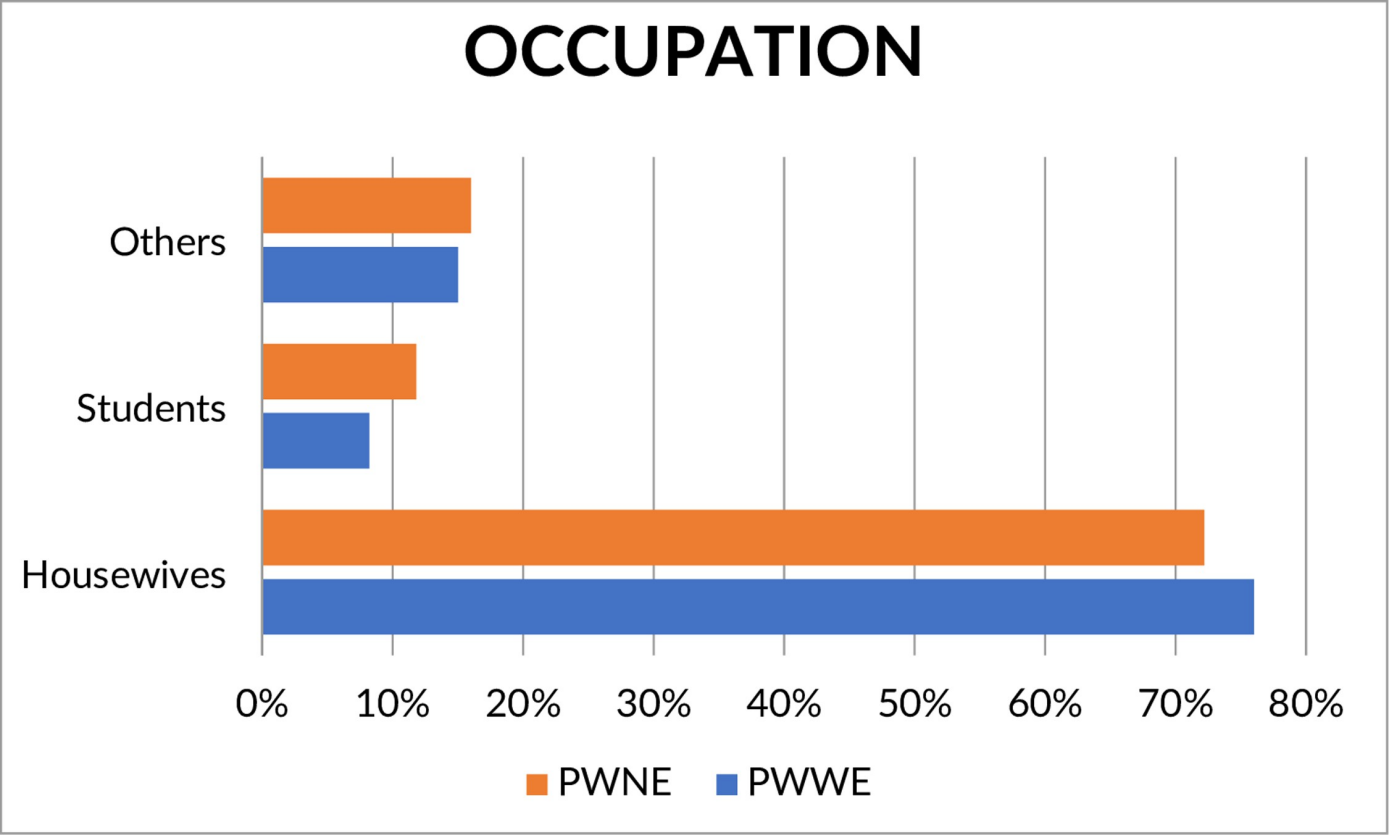
Comparison of professional occupations between pregnant women with (PWWE) and without epilepsy (PWNE). The figure shows that pregnant women without epilepsy are students and have professional occupations while most pregnant women with epilepsy are housewives.

Most women were housewives (PWWE [n 17 = 0/221, 76.9%], PWNE [n = 353/489, 72.2%]), followed by students (PWWE [n = 18/221, 8.1%], PWNE [n = 58 / 220, 11.8%]), and those who had another profession (PWWE [n = 33/220, 14.0%], PWNE [n = 78/489, 16%], *p* = 0.28) (Fig 2 and S1 Table). No association was observed between PWWE and unemployment (adjusted OR = 1.46, 95% CI = 0.83–2.58, *p* = 0.18) (Table 3).

**Table 3:** Analysis of crude and age-adjusted odds ratio of socio-demographic variables of pregnant women with epilepsy.

Variable	OR crude			OR adjusted by age		
	OR*	95% CI	*p*-value	OR adjusted	95% CI	p-value
**Occupation**						
Students	Reference	-	-	Reference	-	-
Housewives	1.61	0.71; 3.66	0.24	1.46	0.83; 2,58	0.18
Others	0.87	0.32; 2.33	0.79	1.27	0.65; 2,50	0.47
**Marital status**						
Single	1.24	0.66; 2.32	0.49	0.93	0.59; 1,47	0.78
Married	Reference	-	-	Reference		-
Stable union	0.68	0.35; 1.29	0.23	0.54	0.33; 0.86	0.01
Divorced/widow	1.94	0.35; 10.45	0.44	1.11	0.29; 4,14	0.87

Most PWWE were single (n = 104/220, 47.3%), but no association was found (adjusted OR = 0.93, 95% CI 0.59–1.47, (*p* = 0,78). Those without epilepsy had an association with stable union (OR adjusted = 0.54, 95% CI 0.33–0.86); *p* = 0.01 (Table 3).

Regarding the number of deliveries, most PWWEs and PWNEs were multiparous, (n = 132/219, 60.3%) and (n = 284/489, 58.1%), respectively (*p* = 0.58) (Tables 1 and S1). No association between PWWE and multiparity was observed after adjustment for age (adjusted OR = 1.03, 95% CI = 0.74–1.44, *p* = 0.82) (Table 4).

**Table 4:** Analysis of crude and age-adjusted odds ratio of obstetric outcomes variables of pregnant women with epilepsy.

Variable	OR crude			OR adjusted by age		
	OR*	CI 95%	*p*-value	OR adjusted	CI 95%	*p*-value
**Previous birth**						
Primipara	Reference	-	-	Reference	-	-
Multipara	0.93	0.58; 1.51	0.78	1.03	0.74; 1.44	0.82
**Delivery**						
Vaginal	Reference	-	-	Reference	-	-
Cesarean	21.83	13.78; 34.57	<0.01	22	14.35; 33.73	<0.01
**Miscarriage**						
No	Reference	-	-	Reference	-	-
Yes	1.52	0.81; 2.89	0.19	1.72	1.13; 2.61	0.01

Pregnant women who had epilepsy were approximately 20 times more likely to have a cesarean delivery than PWNE (adjusted OR = 22.0, 95% CI = 14.35–33.73, *p* < 0.01), with a positive association between PWWE and abortion (adjusted OR = 1.72; 95% CI = 1.13–2.61, *p* = 0.01) (Table 4).

## Discussion

This research is pioneer in the analysis of the socio-demographic profile of PWWE in the Northeast region of Brazil. Regarding the characteristics of these women, the results showed that most of them lived rural areas, were illiterate, had cesarean delivery at childbirth, and had at least one abortion. Considering both groups, PWWE and PWNE, we observed that they were mostly brown-colored, single, housewives, and multiparous.

The prevalence of PWWE (0.49%) between the years 2008 and 2020 in high-risk pregnancy referral centers of Alagoas state is in agreement with other authors, who described a prevalence of 0.4% to 0.8% in this population [8]. Our results showed that more than half of PWWE live in the interior of the state, where only 26.36% of the Alagoas population resides [29] and that they are more likely to have epilepsy than pregnant women who were born in the capital. Epilepsy is a global disease with uneven distribution. Approximately 80% of affected individuals reside in low- and middle income countries. The incidence and prevalence of epilepsy in low-income populations are higher than those in the rest of the world. The average lifetime prevalence of epilepsy in developed countries is 5.8 per 1,000, while in rural areas of developing countries it is 15.4 per 1,000 [6]. The severe precariousness of resources in the interior of the Alagoas state may be the reason of the higher percentage of PWWE found in this location and may also explain the younger age at which WWE become pregnant compared to other countries. In contrast to this study, one study conducted in the city of Toledo, Paraná State, which included both men and WWE, found that 83.8% of them were from urban areas, where 77.38% of the population is concentrated [30].

This study found no age difference between the PWWE and PWNE. Our sample was also younger than samples of PWWE from studies conducted in other countries, where the average age ranged between 27.4 and 30.4 years old [7–29]. Between 2012 and 2016, the Brazilian Northeast region was identified as the third with the highest number of hospitalizations of WWE in this reproductive group (n = 8,245, 19.53%). Among them, the most frequent were women between 20 and 29 years old (n = 1,944, 20.32%) [12].

Regarding race, the majority of pregnant women with and without epilepsy were of brown ethnicity (88.6%), which is in line with previous work conducted in the Brazilian Northeast region, which showed that 49.22% of hospitalized WWE were brown-colored [12]. The population in Alagoas between 2012 and 2019 showed that 44.87% were brown, favoring this high rate among pregnant women [29].

Concerning the type of delivery, PWWE were approximately 20 times more likely to have a cesarean section than PWNE. This study conducted in the United States also verified these results, albeit with a smaller probability [10]. Consistent with our results, research studies performed in the US and China observed that PWWE were more likely than PWNE to have cesarean delivery (40.5% vs. 33.1% and 85.3% vs. 50.3%, respectively) [10–31]. Previous studies have concluded that PWWE have an increased risk of preterm delivery, cesarean section, and labor induction during childbirth [11–31]. Other authors have described that factors such as multiparity, age, and probable complications in childbirth contribute to cesarean section, in addition to the need for tubal ligation [32]. In contrast to our results, European studies found lower cesarean delivery rates in PWWE (18.8% vs. 34%) [9–26]. Additionally, some studies reported a higher incidence of vaginal deliveries [33, 34]. Brazilians seem to have a culture concerning their preference for cesarean section delivery, where social, institutional, financial, and obstetric practice factors influence their choice [32]. In agreement with this information, the cesarean rate in the state of Alagoas was 52.3% in 2019 [29], which is higher than that observed in European studies, but smaller than that observed in our patients with epilepsy.

Another observation was between PWWE and the occurrence of spontaneous abortion, which was significantly different from that observed among PWNE. The values of miscarriages in epileptic women in our research and in those of other studies were (21.9%), (21.4%) and (8.5%), respectively [24–28]. An American study also showed consonant results (OR adjusted, 1.27; 95% CI, 1.17–1,38) [10]. The Australian Register between 1999 to end 2014 and a meta-analysis of 39 studies from 1990 to 2015, comprising 2,837,325 pregnancies in low- and high-income countries, compared PWWE versus PWNE to look the association between epilepsy in pregnancy as well as exposure to AEDs and several maternal and fetal outcomes. It concluded that PWWE is associated with adverse pregnancy outcomes, such as miscarriage (OR 1.54; 95% CI 1.02–2.32; 67%) (OR = 1,45; 95% CI1,12–1,87; 21,4%) [23, 24].

In this study, multiparity was evident in both PWWE and PWNE (58%–60%), which is in contrast with other studies. A recent Brazilian study in Toledo city, Paraná, showed multiparity rates of 15,4% among WWE [30]. European studies found rates between 20.8% and 36% for multiparity [7–26], while a study performed in the United States observed very low rates among PWWE and PWNE (1,7%–1,8%) [10]. In contrast with our study with our study the population-based retrospective cohort in Sweden, a higher number of primiparous patients were found [27].

Considering the findings of our study and the socio-demographic profile of PWE in low- and middle-income countries, it would be expected to observe lower levels of education and higher levels of unemployment among PWWE from the Alagoas state than in other regions, characterized by strong economic resources. Regarding employment, no association was observed between PWWE and unemployment; however, a similar and high number of PWWEs and PWNEs reported being homemakers, which may be in part related to the unemployment.

Regarding education, we verified that PWNEs were significantly more literate than PWWE, and an association between these groups of women with illiteracy was found. Multiparity, low education, and unemployment may be highly related. According to studies, 50% of girls in Teresina, a city in northeastern Brazil, stopped their studies during their first pregnancy [35], which may have a serious influence on the probability of having a job and contribute to the unemployment rate. The same hypothesis may be applied to our population for explaining the observed results. In addition, the Brazilian National Survey by Samples by Household of 2015 (PNAD) has shown severe per capita inequality in the country and the lowest average income of the northeastern population as well as in other populations [36]. To assist the underprivileged population, the Brazilian government through social inclusion programs, such as the Family Allowance Program, performs monthly income transfers to poor and extremely poor families [37]. Such programs have assisted and addressed the basic needs of these populations; however, they may have also contributed to the high number of women multiparity rates WWE [37].

According to previous studies, the socio-demographic profile of PWE living in low- and middle-income countries is characterized by low education and unemployment [6]. However, such findings are not exclusive to less-developed countries, since a study involving seven European countries reported low levels of education among PWE who attained elementary or secondary levels. In addition, 18% of them were unemployed [38]. Considering the national panorama, a study from Paraná (South Brazilian region) found that the majority of PWE had incomplete primary education (59.6%), while only 14.0% completed primary school [30], which is in line with the results of our work. A contrast may be observed between the results observed in Santa Catarina [22], another South Brazilian region, where, despite the small sample, the authors observed that 30.2% (n = 13) of PWE were illiterate, while in our PWWE sample, 7% (n = 15) had no schooling. In line with that result, the present study revealed that being a homemaker was the main occupation among PWWE (76.9%), while only 8.2% were students, thus confirming the low levels of education and high levels of unemployment. However, we also found low levels of education (11.8%) and unemployment (72.2%) in pregnant women without epilepsy, perhaps because the high-risk pregnancy centers where the study sample was recruited are public institutions.

Regarding the composition of the family nucleus, our results did not show a significant difference in marital status between PWWE and PWNE, and indicated that most PWWE were single (47.3%), while most PWNE had stable union (43.8%). Our study found no association between PWWE and marital status. Previous studies conducted in European countries and in the southern Brazilian region described the stigma of marriage in PWE, showing that this population is more likely to be single or divorced [38, 39], while others described them as being mostly single (46.5%) and married or in a stable relationship (44.2%) [22].

## Conclusion

The aims were achieved, data from this research on PWWE from Alagoas suggests adverse outcomes in obstetrics are associated with higher cases of cesarean delivery and miscarriage. The socio-demographic profile shows the predominance of illiteracy and residing of the interior of state, characterized by precarious economic resources. The results of this work demonstrate that PWWE experience conditions of vulnerability.

## Supporting information

S1 Table Comparison of average socio-demographic and obstetric outcome variables between pregnant women with and without epilepsy. DOI: 10.21203/rs.3.rs-1523664/v1.
(PDF)

## List of abbreviations

PWE: people with epilepsy
WWE: women with epilepsy
PWWE: pregnant women with epilepsy
PWNE: pregnant women without (no) epilepsy
AED: antiepileptic drugs
