# Supplementary material for: Socio-demographic profiles and obstetrics outcomes of pregnant women with epilepsy in a vulnerability State, Brazil
Source: PLoS One. 2022 Jul 20;17(7):e0271328. doi: 10.1371/journal.pone.0271328 (PMC9299315; doi:10.1371/journal.pone.0271328)
Supplement: S1 Table — DOI: 10.21203/rs.3.rs-1523664/v1. (PDF) [file pone.0271328.s001.pdf]

S1 Table. Comparison of average and *p* value in sociodemographic and obstetrics outcomes variables between pregnant women with and without epilepsy

**Variables**	**Pregnant with Epilepsy (n = 224)**	**Pregnant without Epilepsy (n = 492)**	**p-value**
**Age**			0,07
**Mean and standard deviation**	24,94 (± 6,25)	23,98 (± 6,89)	
**Origin**			< 0,01
Maceio (n = 351)	92 (41,8%)	259 (52,7%)	
Countryside (n = 360)	128 (58,2%)	232 (47,3%)	
total	220(100%)	491(100%)	
**Race/color**			0,17
White (n = 62)	15 (6,8%)	47 (9,7%)	
Black (n = 22)	10 (4,6%)	12 (2,5%)	
Brown (n = 620)	195 (88,6%)	425 (87,8%)	
Total	220(100%)	484(100%)	
**Education**			< 0,01
Illiteracy (n = 29)	15 (7,0%)	14 (3,0%)	
Fundamental complete or incomplete (n = 396)	112 (52,1%)	284 (60,7%)	
High school complete or incomplete (n = 245)	80 (37,2%)	165 (35,3%)	
Graduation complete or incomplete (n = 13)	8 (3,7%)	5 (1,0%)	

Total	215(100%)	468(100%)	
**Marital status**			< 0,01
Single (n = 297)	104 (47,3%)	193 (39,5%)	
Married (n = 122)	46 (20,9%)	76 (15,5%)	
Stable union (n = 280)	66 (30%)	214 (43,8%)	
Divorced/ widow (n = 10)	4 (1,8%)	6 (1,2%)	
Total	220(100%)	489(100%)	
**Occupation**			0,28
Housewives (n = 523)	170 (76,9%)	353 (72,2%)	
Students (n = 76)	18 (8,2%)	58 (11,8%)	
Others (n = 111)	33 (14,9%)	78 (16,0%)	
**Total**	221(100%)	489(100%)	
**Previous birth**			0,58
Primipara (n = 292)	87 (39,7%)	205 (41,9%)	
Multiparous (n = 416)	132 (60,3%)	284 (58,1%)	
**Total**	219(100%)	489(100%)	
**Delivery**			< 0,01
Vaginal (n = 482)	52 (25,4%)	430 (87,4%)	
Cesarean (n = 215)	153 (74,6%)	62 (12,6%)	
Total	205(100%)	492(100%)	
**Miscarriages**			< 0,01
No (n = 594)	171 (78,1%)	423 (86,5%)	
Yes (n = 114)	48 (21,9%)	66 (13,5%)	
Total	219(100%)	489(100%)	

*Five participants of the indigenous race were excluded from the analyses, so that the tests could be carried out.
